# Tumor suppressor p53 negatively regulates glycolysis stimulated by hypoxia through its target RRAD

**DOI:** 10.18632/oncotarget.2137

**Published:** 2014-06-26

**Authors:** Cen Zhang, Juan Liu, Rui Wu, Yingjian Liang, Meihua Lin, Jia Liu, Chang S. Chan, Wenwei Hu, Zhaohui Feng

**Affiliations:** ^1^ Department of Radiation Oncology, Rutgers Cancer Institute of New Jersey, Rutgers, State University of New Jersey, New Brunswick, USA; ^2^ Department of Medicine, Rutgers Cancer Institute of New Jersey, Rutgers, State University of New Jersey, New Brunswick, USA

**Keywords:** p53, glycolysis, RRAD, hypoxia, lung cancer

## Abstract

Cancer cells display enhanced glycolysis to meet their energetic and biosynthetic demands even under normal oxygen concentrations. Recent studies have revealed that tumor suppressor p53 represses glycolysis under normoxia as a novel mechanism for tumor suppression. As the common microenvironmental stress for tumors, hypoxia drives the metabolic switch from the oxidative phosphorylation to glycolysis, which is crucial for survival and proliferation of cancer cells under hypoxia. The p53's role and mechanism in regulating glycolysis under hypoxia is poorly understood. Here, we found that p53 represses hypoxia-stimulated glycolysis in cancer cells through RRAD, a newly-identified p53 target. RRAD expression is frequently decreased in lung cancer. Ectopic expression of RRAD greatly reduces glycolysis whereas knockdown of RRAD promotes glycolysis in lung cancer cells. Furthermore, RRAD represses glycolysis mainly through inhibition of GLUT1 translocation to the plasma membrane. Under hypoxic conditions, p53 induces RRAD, which in turn inhibits the translocation of GLUT1 and represses glycolysis in lung cancer cells. Blocking RRAD by siRNA greatly abolishes p53's function in repressing glycolysis under hypoxia. Taken together, our results revealed an important role and mechanism of p53 in antagonizing the stimulating effect of hypoxia on glycolysis, which contributes to p53's function in tumor suppression.

## INTRODUCTION

Metabolic alterations are a hallmark of cancer cells [1-3]. Unlike normal cells that mainly depend upon oxidative phosphorylation to provide energy, cancer cells preferentially utilize glycolysis even under normal oxygen concentrations (normoxia). This enhanced aerobic glycolysis in cancer cells is known as the Warburg effect, which is characterized by dramatically increased rates of glucose uptake and utilization in cancer cells than normal cells [1-3]. Recent studies strongly suggest that the enhanced aerobic glycolysis can exert fundamental effects on tumor cell proliferation and survival [2-4].

Tumor suppressor p53 plays a critical role in suppressing the initiation and/or development of tumors. In response to stress, p53 regulates the transcription of its target genes and initiates various cellular responses, including cell cycle arrest, apoptosis and senescence, to prevent tumorigenesis [5-8]. Recent studies have revealed that p53 plays a critical role in negative regulation of the aerobic glycolysis in cancer cells [9-12]. For instance, p53 was reported to repress the aerobic glycolysis by inducing SCO2 [13] and TIGAR [14] under normoxic conditions.

Hypoxia is a common feature of malignancy and particularly of solid tumors [15-17]. Under hypoxic conditions cancer cells develop an efficient adaptive metabolic response to ensure their survival and proliferation. It has been well-established that hypoxia can drive the metabolic switch from the oxidative phosphorylation to glycolysis, which is critical for the survival and proliferation of cancer cells in a hypoxic environment [15-17]. Although hypoxia-inducible factor-1 (HIF-1) has been reported to contribute greatly to this enhanced glycolysis under hypoxia through transcriptional activation of genes related in glycolysis, the precise mechanisms controlling metabolism and cell growth under hypoxia are not fully understood [15-17]. As a key negative regulator of glycolysis under normoxic conditions, the role of p53 in regulation of the glycolysis under hypoxic conditions and its underlying mechanism is poorly understood.

In this study, p53 was found to play an important role in negative regulation of glycolysis under hypoxia through induction of RRAD (Ras-related associated with diabetes), the Ras-related small GTPase. RRAD was first identified as a gene overexpressed in some Type II diabetic patients [18]. It was reported that RRAD overexpression reduced insulin-stimulated glucose uptake in cultured cells from mouse muscle and fat, two main insulin-responsive tissues. However its underlying mechanism is unclear [19]. Recently, RRAD was identified as a gene regulated by p53 in response to chemotherapeutic agents, and can mediate p53's function in inhibition of tumor cell migration [20]. Furthermore, RRAD expression is frequently diminished in human cancer, including lung and breast cancer, which is associated with tumor progression and poor prognosis in cancer patients [20, 21]. Results from this study showed that p53 induced RRAD expression under hypoxia, which in turn negatively regulated glycolysis driven by hypoxia through inhibition of the translocation of glucose transporter 1 (GLUT1) to the plasma membrane of cells. Thus, the results from this study revealed an important role and mechanism for p53 to maintain homeostasis of glucose metabolism under hypoxic conditions, which contributes to p53's role in tumor suppression.

## RESULTS

### p53 negatively regulates glycolysis enhanced by hypoxia

**Figure 1:**
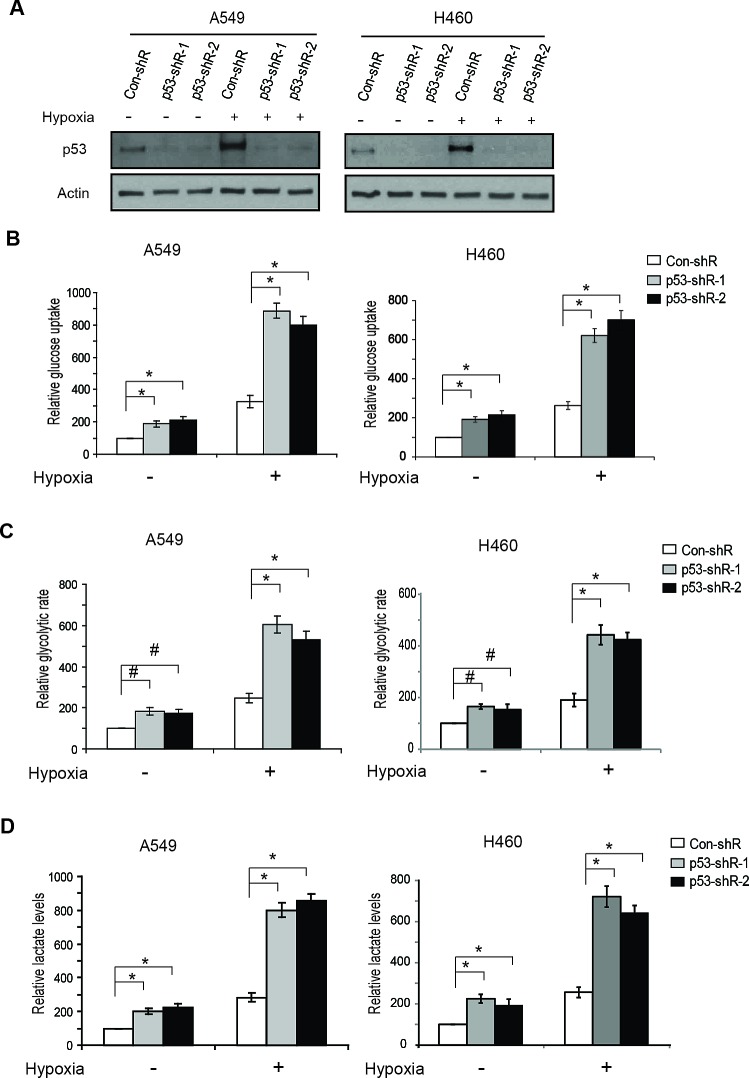
p53 reduces glucose uptake, the glycolytic rate and lactate production in human lung cancer cells under hypoxic conditions (A) Hypoxia activated p53 in human lung A549 and H460 cells. The p53 wild-type A549-con-shR and H460-con-shR cells, as well as A549-p53-shR and H460-p53-shR cells with stable p53 knockdown by shRNA vectors were treated with hypoxia (0.1% O_2_) for 24 h before assays. Two different shRNA vectors against p53 were employed. The levels of p53 were measured by Western-blot assays. (B-D) Knockdown of p53 increased the glucose uptake (B), the glycolytic rate (C) and lactate production (D) in A549 and H460 cells under both normoxic and hypoxic conditions. Data are presented as mean value ± SD (n=3). #: *p*<0.05; * *p*<0.01 (Student's *t* tests).

Recently, p53 has been reported to play a critical role in repressing the aerobic glycolysis under normoxic conditions through its transcriptional regulation of several target genes, including SCO2, TIGAR [13, 14]. As a common feature of solid tumors, hypoxia is a driving force to switch cellular metabolism from mitochondrial oxidative phosphorylation to glycolysis [15-17]. However, the role of p53 in regulating glycolysis under hypoxic conditions is poorly understood. To investigate how p53 regulates glycolysis in cells under hypoxia, A549 and H460, two human lung cancer epithelial cell lines expressing wild-type p53 were employed. These cell lines were stably transduced with either shRNA retroviral vectors to knock down the endogenous p53 (A549-p53-shR and H460-p53-shR) or control vectors (A549-con-shR and H460-con-shR) (Figure 1A). These cells were then treated with hypoxia and the levels of glucose uptake, glycolytic rate, and lactate production of these cells were measured. Hypoxia greatly stimulated glucose uptake (Figure 1B), the glycolytic rate (Figure 1C), and lactate production (Figure 1D) in the control cell lines expressing wild-type p53 as well as the cell lines with stable p53 knockdown. Notably, p53 activation in response to hypoxia (Figure 1A) greatly reduced the stimulating effect of hypoxia on glycolysis in the cells; under hypoxic conditions much lower levels of glucose uptake, glycolytic rate and lactate production were observed in A549-con-shR and H460-con-shR compared with A549-p53-shR and H460-p53-shR cells, respectively (Figure 1B-D). This inhibitory effect of p53 under hypoxic conditions is consistent with the reported role of p53 in negative regulation of glucose uptake, glycolytic rate, and lactate production under normoxic conditions (Figure 1B-D). These results clearly showed that p53 plays a critical role in antagonizing the stimulating effect of hypoxia on glycolysis to maintain the homeostasis of glucose metabolism in cells.

### RRAD negatively regulates glycolysis

RRAD was first identified as a gene overexpressed in some Type II diabetic patients [18]. RRAD overexpression was reported to reduce insulin-stimulated glucose uptake in cultured cells from mouse muscle and fat, although its mechanism is unclear [19]. Recent studies showed that RRAD expression is frequently down-regulated in human lung cancer, which is associated with tumor progression and poor prognosis in cancer patients [20, 21]. These findings suggest a potential role of RRAD in regulation of glucose metabolism in lung cancer.

**Figure 2:**
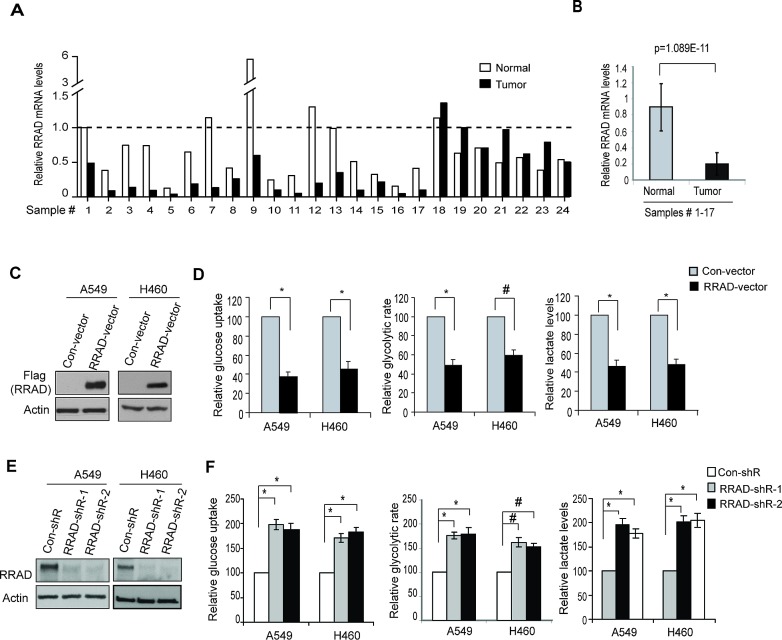
RRAD negatively regulates glucose uptake, the glycolytic rate and lactate production in human lung cancer cells (A) The relative RRAD mRNA expression in human lung cancer samples and their matched adjacent non-tumor tissues (n=24). The mRNA levels of RRAD were measured in 24 human lung cancer samples and their matched adjacent non-tumor tissues (Origene, Rockville) by Taqman real-time PCR assays. The mRNA levels of RRAD were normalized with Actin. The relative RRAD mRNA level in the adjacent non-tumor tissue of the cancer sample #1 was designated as 1. (B) The relative mean value of RRAD mRNA in human lung cancer samples #1-17 and their matched adjacent non-tumor lung tissues. Data are presented as mean value ± SD (n=17). *P*=1.089E-11. (C) Western-blot analysis of the ectopic RRAD expression in A549 and H460 cells stably transduced with pLPCX-RRAD-Flag retroviral vectors (RRAD) or control vectors (Con). (D) Ectopic RRAD expression reduced glucose uptake, the glycolytic rate and lactate production in A549 and H460 cells. (E) RRAD knockdown by shRNA in A549 and H460 cells detected by Western-blot assays. Cells were stably transduced with 2 different shRNA vectors against RRAD (RRAD-shR) or control shRNA (Con-shR). (F) Knockdown of endogenous RRAD by shRNA increased the glucose uptake, the glycolytic rate and lactate production in A549 and H460 cells. Data are presented as mean value ± SD (n=3). #: *p*<0.05; * *p*<0.01 (Student's *t* tests).

To investigate the potential role of RRAD in glycolysis in lung cancer, we first examined the RRAD expression in lung cancer samples and their matched adjacent non-tumor lung tissues (n=24; provided by Origene). RRAD mRNA levels were significantly reduced (by ~4-fold) in a high percentage of lung cancer samples we analyzed (17/24) compared with their matched adjacent normal lung tissues (*p*=1.089E-11; Figure 2A, B), which is consistent with previous reports [20, 21]. To investigate whether the decreased RRAD expression contributes to enhanced glycolysis in lung cancer, RRAD was stably overexpressed by a RRAD expression vector or knocked down by shRNA vectors in A549 and H460 cells. Ectopic expression of RRAD in both cell lines clearly reduced glucose uptake, the glycolytic rate and lactate production (Figure 2C, D), whereas knockdown of endogenous RRAD by 2 different shRNA vectors both clearly increased glucose uptake, the glycolytic rate and lactate production (Figure 2E, F). These results demonstrated that RRAD negatively regulates glycolysis in lung cancer cells under nomoxic conditions, suggesting that the diminished RRAD expression is a novel mechanism contributing to the enhanced aerobic glycolysis (the Warburg effect) in lung cancer.

### RRAD inhibits the translocation of GLUT1 to the plasma membrane

**Figure 3:**
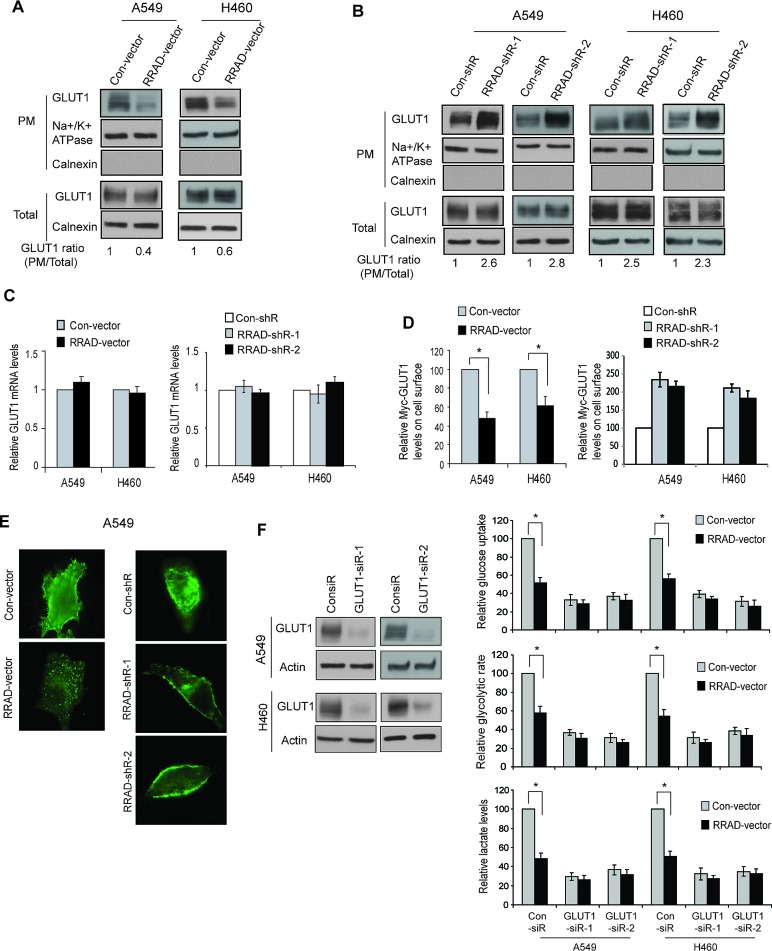
RRAD negatively regulates GLUT1 translocation to the plasma membrane (PM) in cells (A) Ectopic RRAD expression reduced GLUT1 translocation to the PM in A549 and H460 cells as analyzed by Western-blot assays. The PM protein Na^+^/K^+^ ATPase was used as an internal control. The ER membrane protein Calnexin was used to exclude the contamination of PM by the other membrane fractions. (B) RRAD knockdown by shRNA promoted GLUT1 translocation to the PM in A549 and H460 cells. (C) Ectopic expression of RRAD or knockdown of endogenous RRAD did not change the mRNA levels of GLUT1 in A549 and H460 cells. The mRNA levels of GLUT1 were measured by Taqman real-time PCR, and normalized with Actin. (D) Ectopic RRAD expression reduced Myc-GLUT1 translocation to cell surface (left panel), whereas RRAD knockdown by shRNA promoted Myc-GLUT1 translocation to cell surface (right panel) in A549 and H460 cells analyzed in a flow cytometer. Relative Myc-GLUT1 levels on cell surface were calculated after normalization with the total Myc-GLUT1 levels in cells. Cells were transduced with pLPCX-Myc-GLUT1 vectors or control pLPCX vectors 48 h before assays. (E) Ectopic RRAD expression reduced Myc-GLUT1 translocation to the cell surface (left panels), whereas RRAD knockdown by shRNA promoted Myc-GLUT1 translocation to the cell surface (right panels) in A549 and H460 cells analyzed by IF staining with an anti-Myc antibody. (F) GLUT1 knockdown largely abolished the inhibitory effect of RRAD overexpression on glucose uptake, the glycolytic rate and lactate production in A549 and H460 cells. Cells with stable ectopic RRAD overexpression or control cells were transfected with 2 different GLUT1 siRNA (GLUT1-siR) or control siRNA for 24 h before assays. GLUT1 knockdown was confirmed by Western-blot assays (left panels). Data are presented as mean value ± SD (n=3). * *p*<0.01 (Student's *t* tests).

Transport of glucose across the plasma membrane (PM) of cells is the first rate-limiting step for glucose metabolism, which is mediated by facilitative glucose transporters (GLUTs) [22]. Increased translocation of GLUTs, such as GLUT1 and GLUT4, from the intracellular pool to the PM of cells promotes glucose transport [22-24]. GLUT4 is the main glucose transporter expressed in insulin-responsive fat and muscle tissues, which translocates to the PM in response to the stimulation of insulin to promote the glucose uptake. GLUT1 is ubiquitously expressed in various cells and tissues, and is responsible for constitutive glucose uptake in these tissues and cells [22]. GLUT1 is frequently overexpressed in various cancers, including lung cancer, which can promote glycolysis [3, 25-28]. Interestingly, RRAD clearly reduced the GLUT1 translocation to the PM in cells but did not affect the total GLUT1 levels in A549 and H460 cells (Figure 3A-C). As shown in Figure 3A, ectopic RRAD expression clearly reduced the levels of endogenous GLUT1 protein on the PM but did not affect the total GLUT1 protein levels in cells as measured by Western-blot assays using isolated PM fractions. Consistently, knockdown of endogenous RRAD clearly increased the levels of endogenous GLUT1 on the PM but not the total GLUT1 levels in cells (Figure 3B). Results from real-time PCR assays confirmed that RRAD did not change the GLUT1 expression at the mRNA level (Figure 3C).

To further confirm our findings, A549 and H460 cells were transduced with pLPCX-Myc-GLUT1 vectors expressing GLUT1 with Myc tag in its first exofacial loop, and the levels of Myc-GLUT1 on the cell surface or in the whole cell were measured by immunofluorescence (IF) staining with an anti-Myc antibody followed by analysis in a flow cytometer. Ectopic expression of RRAD clearly reduced the levels of Myc-GLUT1 protein on the PM but did not affect the total Myc-GLUT1 levels in A549 and H460 cells (Figure 3D). Consistently, knockdown of RRAD clearly increased the levels of Myc-GLUT1 on the PM but did not affect the total Myc-GLUT1 levels in the cells (Figure 3D). Similar results were observed when the cells transduced with pLPCX-Myc-GLUT1 vectors were stained with the anti-Myc antibody and observed under a confocal microscope; ectopic expression of RRAD clearly reduced the translocation of Myc-GLUT1 protein from cytoplasm to the cell surface in A549 cells (Figure 3E, left panels), whereas RRAD knockdown promoted the translocation of Myc-GLUT1 protein from cytoplasm to the cell surface in the cells (Figure 3E, right panels).

To investigate whether inhibition of GLUT1 translocation mediates the role of RRAD in negative regulation of glycolysis in cells, GLUT1 was knocked down by two different siRNA oligos in A549 and H460 cells with and without ectopic RRAD expression (Figure 3F, left panel). GLUT1 knockdown clearly reduced the glucose uptake, the glycolytic rate and lactate production in both A549 and H460 cells. Notably, GLUT1 knockdown largely abolished the inhibitory effect of RRAD on glycolysis in both cell lines; ectopic expressed RRAD showed much less inhibitory effect upon glucose uptake, the glycolytic rate and lactate production in cells with GLUT1 knockdown compared with control cells without GLUT1 knockdown (Figure 3F, right panels). Taken together, these results demonstrated that RRAD inhibits the GLUT1 translocation to the PM, which is an important mechanism for RRAD to repress the aerobic glycolysis in cancer cells.

### p53 induces RRAD expression under hypoxic conditions

**Figure 4:**
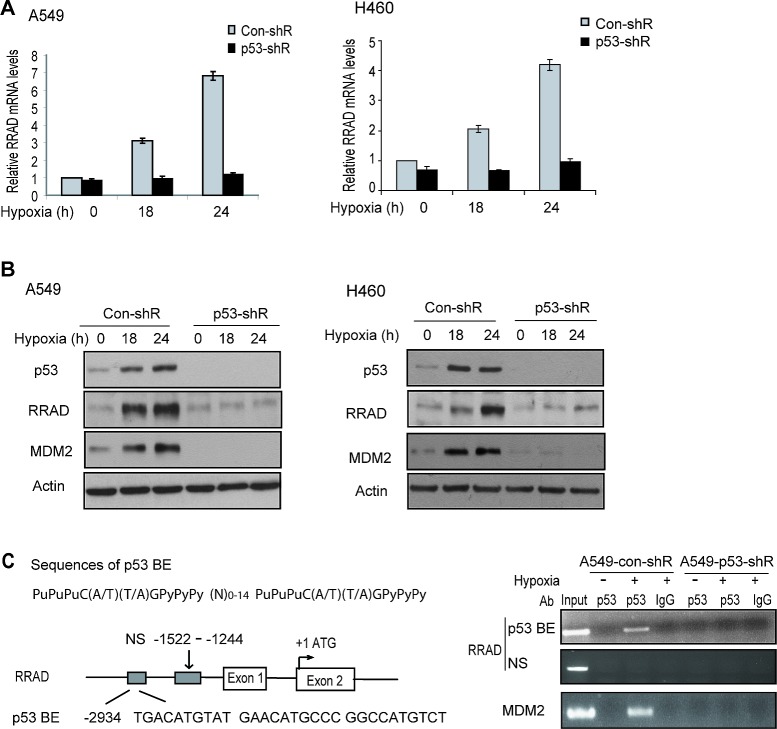
p53 induces RRAD expression under hypoxic conditions (A, B) Activated p53 induced RRAD expression at both mRNA (A) and protein (B) levels under hypoxic conditions in A549 and H460 cells. A549-con-shR, A549-p53-shR, H460-con-shR and H460-p53-shR cells were treated with hypoxia for 18 or 24 h before mRNA and protein levels of RRAD were measured by real-time PCR (A) and Western-blot assays (B), respectively. The mRNA levels of RRAD were normalized with actin. p53 target MDM2 was used as a positive control for p53 activation. Two different p53 shRNA vectors were used and very similar results were obtained. (C) p53 bound to the p53 DNA binding element (p53 BE) in the RRAD promoter under hypoxic conditions detected by ChIP assays. Upper left panel: The consensus DNA sequences for the p53 BE. N, any nucleotide; Pu, purine; Py, pyramidine. Lower left panel: The p53 BE in human RRAD promoter. Number indicates the nucleotide position relative to the ATG site (+1). Right panel: ChIP analysis with the p53 antibody (DO-1) in A549-con-shR and A549-p53-shR cells treated with hypoxia for 18 h. The non-specific (NS) DNA fragment in the RRAD promoter (−1522 to −1244) which does not contain any potential p53 BE was used as a negative control. The p53 BE in MDM2 promoter served as a positive control.

As a transcription factor, p53 mainly exerts its function through selectively transcriptional regulation of its target genes in response to different stress signals. SCO2 and TIGAR were identified as genes that are upregulated by p53 under normoxic conditions and contribute to the role of p53 in repressing aerobic glycolysis in cells [13-14]. However, no clear p53-dependent induction of SCO2 or TIGAR was observed in A549 and H460 cells treated with hypoxia (Supplemental Figure S1). Interestingly, RRAD were clearly induced in A549-con-shR but not A549-p53-shR cells treated with hypoxia at both mRNA and protein levels (Figure 4A, B, left panels). This p53-dependent induction of RRAD by hypoxia was confirmed in H460-con-shR and H460-p53-shR cells (Figure 4A, B, right panels). Results from chromatin immunoprecipitation (ChIP) assays further showed that the DO-1 p53 antibody specifically pulled down the DNA fragment containing the p53 DNA binding element (p53 BE) in the RRAD promoter in A549-con-shR cells but not in A549-p53-shR cells treated with hypoxia (Figure 4C), indicating that the activated p53 protein can bind to the p53 BE in the RRAD promoter in response to hypoxia to directly activate RRAD transcription under hypoxic conditions.

### RRAD mediates the role of p53 in repressing glycolysis under hypoxic conditions

**Figure 5:**
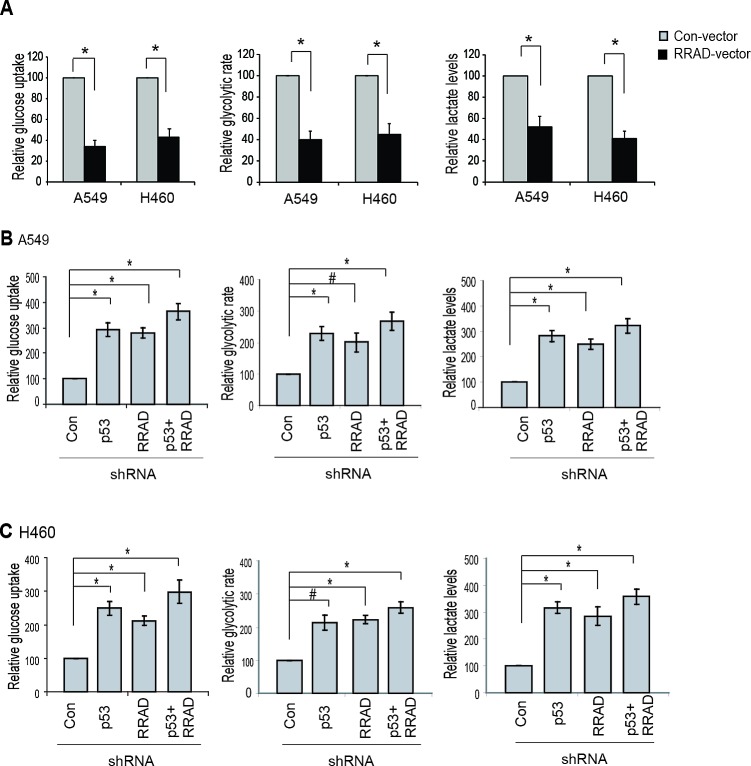
RRAD mediates p53's function in negative regulation of glycolysis under hypoxic conditions (A) Ectopic RRAD expression reduced glucose uptake, the glycolytic rate and lactate production stimulated by hypoxia in A549 and H460 cells. Cells with stable ectopic RRAD expression or control cells were treated with hypoxia for 24 h before assays. (B, C) RRAD mediated p53's function in repressing glucose uptake, the glycolytic rate and lactate production stimulated by hypoxia in A549 (B) and H460 (C) cells. Cells transduced with control or RRAD shRNA vectors together with or without p53 shRNA vectors were treated with hypoxia for 24 h before assays. Data were presented as mean value ± SD (n=3). #: *p*<0.05; * *p*<0.01 (Student's *t* tests).

Our findings that RRAD can repress glycolysis under normoxic conditions and that RRAD can be induced by p53 in response to hypoxia strongly suggest that RRAD may mediate the role of p53 in repressing glycolysis under hypoxic conditions. Here, we investigated whether RRAD contributes to the p53's function in repressing glycolysis stimulated by hypoxia in A549 and H460 cells. Ectopic expression of RRAD in A549 and H460 cells greatly reduced glucose uptake, the glycolytic rate, and lactate production under hypoxic conditions (Figure 5A), demonstrating an important role of RRAD in antagonizing the stimulating effect of hypoxia on glycolysis. Notably, while individual knockdown of RRAD or p53 by shRNA vectors in A549 and H460 cells significantly promoted glucose uptake, the glycolytic rate, and lactate production under hypoxic conditions, RRAD knockdown in A549-p53-shR and H460-p53-shR cells only slightly enhanced glucose uptake, the glycolytic rate, and lactate production (Figure 5B, C). The promoting effect of simultaneous knockdown of p53 and RRAD on the glucose uptake, glycolytic rate and lactate production was much less than the anticipated additive effects of individual knockdown of p53 and RRAD, respectively. These results suggest that RRAD is an important mediator for p53 to antagonize the stimulating effect of hypoxia on glycolysis; in response to hypoxia, p53 activation induces RRAD expression, leading to the down-regulation of glycolysis in lung cancer cells.

### RRAD mediates p53's function in inhibiting GLUT1 translocation under hypoxic conditions

**Figure 6:**
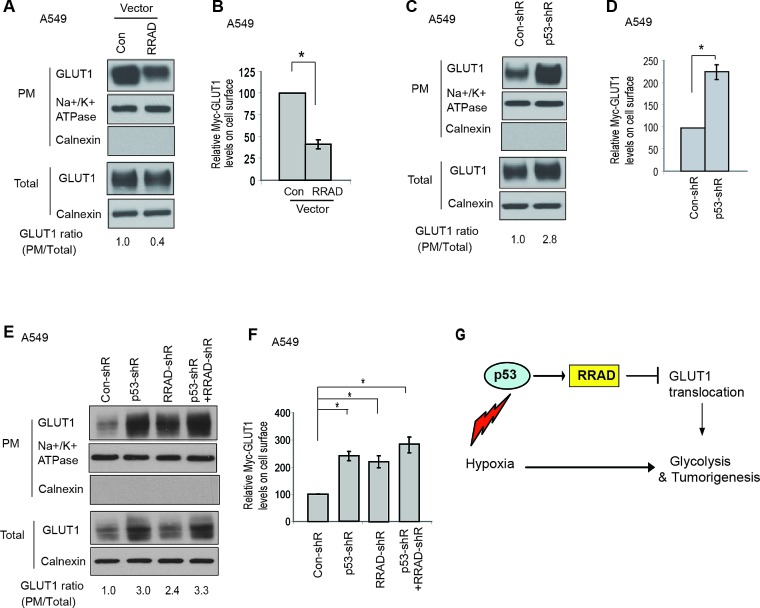
p53 negatively regulates GLUT1 translocation to the plasma membrane (PM) through RRAD under hypoxic conditions (A) Ectopic expression of RRAD reduced GLUT1 translocation to the PM under hypoxia in A549 cells as measured by Western-blot analysis. Cells with stable ectopic RRAD expression and control cells were treated with hypoxia for 24 h before assays. (B) Ectopic RRAD expression reduced Myc-GLUT1 translocation to the cell surface in A549 cells under hypoxia as measured by a flow cytometer. Cells were transfected with Myc-GLUT1 vectors and then treated with hypoxia for 24 h before assays. The levels of Myc-GLUT1 on the cell surface were detected by a flow cytometer, and normalized with the total levels of Myc-GLUT1 in cells. (C) p53 inhibited GLUT1 translocation to the PM under hypoxia in A549 cells as measured by Western-blot analysis. A549-con-shR and A549-p53-shR cells were treated with hypoxia for 24 h. (D) p53 inhibited Myc-GLUT1 translocation to the PM under hypoxia in A549 cells. (E, F) RRAD mediated p53's function in inhibition of GLUT1 translocation to the PM under hypoxia. A549-con-shR and A549-p53-shR cells were transduced with RRAD shRNA or control shRNA, followed by hypoxia treatment for 24 h before assays. (E) Western-blot analysis of the levels of endogenous GLUT1 on the plasma membrane. (F) The levels of Myc-GLUT1 on the cell surface measured by a flow cytometer. Data are presented as mean value ± SD (n=3). * *p*<0.01 (Student's *t* tests). (G) Schematic depicting that p53 negatively regulates glycolysis through the RRAD/GLUT1 signaling under hypoxia.

Our finding that RRAD inhibits GLUT1 translocation to the PM under normoxic conditions strongly suggests that RRAD may inhibit GLUT1 translocation under hypoxic conditions, which could be an important mechanism for p53 to repress the glycolysis under hypoxic conditions. Here, we tested whether RRAD can inhibit GLUT1 translocation under hypoxic conditions to mediate p53's function to repress the glycolysis under hypoxic conditions. Indeed, consistent with the role of RRAD in repressing GLUT1 translocation to the PM under normoxic conditions (Figure 3), ectopic expression of RRAD in A549 cells clearly reduced the translocation of both endogenous GLUT1 or Myc-GLUT1 to the PM under hypoxic conditions (Figure 6A, B). p53 was reported to repress the transcription of GLUT1 [29]. As shown in Figure 6C, knockdown of p53 slightly increased total GLUT1 expression in A549 cells under hypoxia. Notably, p53 clearly inhibited GLUT1 translocation to the PM under hypoxic conditions; the PM translocation of both endogenous GLUT1 and Myc-GLUT1 in A549-con-shR cells was much less than that in A549-p53-shR cells (Figure 6C, D). Furthermore, under hypoxia while RRAD knockdown by shRNA in A549-con-shR cells greatly promoted the translocation of both endogenous GLUT1 and Myc-GLUT1 to the PM, RRAD knockdown in A549-p53-shR cells did not further clearly promote the translocation of either endogenous GLUT1 or Myc-GLUT1 (Figure 6E, F). The effect of simultaneous knockdown of p53 and RRAD was much less than the anticipated additive effect of individual knockdown of p53 and RRAD, respectively. Similar results were observed in H460-con-shR and H460-p53-shR cells (Supplemental Figure S2). These results demonstrated that inhibition of GLUT1 translocation is an important mechanism for p53 to repress glycolysis under hypoxic conditions, and this effect can be mediated by the transcriptional induction of RRAD by p53 in response to hypoxia (Figure 6G).

## DISCUSSION

It has been well-established that hypoxia drives the metabolic switch from the oxidative phosphorylation to glycolysis, which plays a critical role in ensuring the survival and proliferation of cancer cells in a hypoxic environment [15-17]. Recent studies have revealed a new function of p53 in negative regulation of aerobic glycolysis in cancer cells under normoxic conditions, which contributes greatly to the role of p53 in tumor suppression [9-11]. However, the role of p53 and its underlying mechanisms in regulation of glycolysis under hypoxic conditions is largely unknown. In this study, we found that p53 plays a critical role in antagonizing the stimulating effect of hypoxia on glycolysis in cancer cells. In response to hypoxia, p53 is activated and binds to the p53 DNA binding element in the promoter of RRAD gene, which in turn activates RRAD transcription. The induction of RRAD by p53 in response to hypoxia leads to the reduced glycolysis through its inhibition of GLUT1 translocation to the PM, which constitutes a novel mechanism for p53 to repress glycolysis stimulated by hypoxia. Considering the critical role of enhanced glycolysis in promoting the survival and proliferation of cancer cells under hypoxia, the role of p53 in antagonizing the stimulating effect of hypoxia on glycolysis in cancer cells should contribute greatly to p53's function in tumor suppression.

It was reported that RRAD was overexpressed in some Type II diabetic patients [18]. RRAD overexpression reduced insulin-stimulated glucose uptake in cultured cells from mouse muscle and fat, two main insulin-responsive tissues. However, its mechanism is unclear [19]. These findings suggest that RRAD overexpression could be involved in the regulation of glucose metabolism in fat and muscle tissues and the development of Type II diabetes. Interestingly, recent findings suggest a potential tumor suppressive function of RRAD in lung cancer. RRAD expression was reported to be down-regulated in many cultured lung cancer cell lines due to RRAD promoter hypermethylation [21]. RRAD expression is frequently down-regulated in human lung cancer, which is associated with poor prognosis in cancer patients [20, 21]. Furthermore, overexpression of RRAD reduces the metastasis of lung cancer cells [20]. Consistent with these reports, we found that RRAD expression is significantly decreased in a high percentage of human lung cancer samples we analyzed compared with their matched adjacent tissues using the cDNA arrays obtained from Origene. Due to the unavailability of genomic DNA for these tumor samples, the methylation patterns of the RRAD promoter and p53 status of the tumor samples are unclear. Importantly, results from this study clearly showed that in lung cancer cells the down-regulation of RRAD expression acts as an important mechanism for cancer cells to promote GLUT1 translocation, and thereby promotes glycolysis under both normoxic and hypoxic conditions. As a p53 target, this function of RRAD contributes significantly to the role of p53 in negative regulation of glycolysis in hypoxic lung cancer cells. Thus, as a newly-identified component of the p53 signaling pathway, RRAD may contribute to the role of p53 in tumor suppression through its regulation of glycolysis. Recently, p53 has been reported to negatively regulate aerobic glycolysis under normoxic conditions through the regulation of many different genes and different mechanisms [9-12]. It is possible that p53 can regulate glycolysis under normoxic conditions through the regulation of different genes and mechanisms in addition to the regulation of RRAD expression and GLUT1 translocation. The crosstalk between HIF and c-Myc has been reported to play a critical role in regulating cancer cell metabolism and promote cancer cell survival and proliferation under hypoxia [30, 31]. It is still not well-understood how p53 regulates cancer cell metabolism through regulating HIF and c-Myc in hypoxic tumor cells. Recently, gain-of-function mutant p53 was reported to promote aerobic glycolysis under normoxic conditions [32]. It is possible that some gain-of-function mutant p53 can promote glycolysis under hypoxic conditions in tumor cells. Future studies will further increase our understanding of how p53 and its gain-of-function mutant forms regulate glycolysis under hypoxia.

Currently, it is still unclear how RRAD inhibits GLUT1 translocation to regulate glycolysis in cells. RRAD was reported to exert many functions through its interaction with other proteins in cells [33-35]. For instance, RRAD was reported to interact with calmodulin and calcium/calmodulin-dependent protein kinase II (CaMKII), and regulate the activity of the pathway [33]. RRAD binds directly to Ca^2+^ channel beta-subunits and regulates voltage-gated calcium channel activity [35]. RRAD was also reported to regulate the expression of certain genes. For instance, RRAD was reported to interact with transcription factor C/EBP-δ and inhibit its binding to the promoter of CTGF (connective tissue growth factor), and thus inhibits the expression of CTGF [36]. Therefore, it is possible that RRAD regulates GLUT1 translocation through interaction with proteins and/or regulation of the expression of proteins that are involved in the regulation of GLUT1 translocation. Future studies will shed further light on the underlying mechanisms by which RRAD regulates GLUT1 translocation.

In summary, our results revealed a novel function of RRAD in repressing glycolysis, and strongly suggest that decreased RRAD expression, which is frequently observed in lung cancer, is an important mechanism contributing to enhanced glycolysis in lung cancer. This study also revealed an important mechanism for p53 to maintain cellular homeostasis of glucose metabolism and prevent glycolysis under hypoxic conditions through its transcriptional up-regulation of RRAD, which in turn inhibits GLUT1 translocation.

## MATERIALS AND METHODS

### Cell culture and plasmids

The p53 wild-type A549 and H460 cells were purchased from ATCC (Manassas, VA). The p53 deficient A549-p53shR and H460-p53shR cells were established by stable transduction with two different pBABE-puro shRNA retroviral vectors against p53 in A549 and H460 cells, respectively, as previously described [37]. The p53 shRNA sequences are as follows: p53-shR-1 (5'-GACTCCAGTGGTAATCTAC-3') and p53-shR-2 (5'-GTCCAGATGAAGCTCCCAGAA-3'). Control cells (A549-con-shR and H460-con-shR) cells were stably transduced with a control pBABE vector. The lentiviral shRNA vectors against human RRAD (V3LHS_364015 and V3LHS_409093) were purchased from Open Biosystems (Huntsville, AL). The siRNA oligos against human GLUT1 (5'- CGAACTATGAACTACAAAGCTTCTA-3', and 5'-TCAAAGTTCCTGAGACTAAAGGCCG- 3') were purchased form Integrated DNA Technologies. siRNA oligos were transfected into cells using Lipofectamine 2000 (Invitrogen). pLPCX-RRAD-Flag vectors were constructed by inserting full length human RRAD cDNA with Flag tag at C-terminus into the pLPCX vectors. For cells with stable ectopic RRAD overexpression, cells were transduced with a pLPCX-RRAD-Flag retroviral vector and selected by puromycin. For hypoxia treatment, cells were treated with hypoxia (0.1% O_2_) in a hypoxia chamber.

### Measurement of glucose uptake, the glycolytic rate and lactate production

Glucose uptake in cells was analyzed as previously described by measuring the uptake of ^3^H-2-deoxyglucose [32, 38]. The glycolytic rate in cells was measured by monitoring the conversion of 5-^3^H-glucose to ^3^H_2_O as previously described [14, 32]. The cell lactate production levels were determined by using a Lactate Assay Kit (Biovision) as previously described [32].

### Western-blot assays

Following antibodies were employed for assays: Anti-RRAD (a generous gift from Dr. CR Kahn, Harvard Medical School); anti-Flag (Sigma); anti-p53 (DO-1; Santa Cruz); anti-MDM2 (2A10); anti-Actin (Sigma); anti-GLUT1 (Abcam); anti-Na^+^/K^+^ ATPase (Novus) and Calnexin (Abcam). Western-blot results were analyzed by using ImageJ 1.45s software (NIH).

### Analysis of endogenous levels of GLUT1 on the PM

The PM fraction of cells was isolated according to standard protocols [32, 39, 40]. Briefly, the PM fraction was separated from the other membrane fraction of cells which includes the endoplasmic reticulum (ER). The expression levels of GLUT1 in the PM fraction were measured by Western-blot assays. A PM protein Na^+^/K^+^ ATPase was detected as an internal standard. Calnexin, an ER membrane protein, was detected to exclude the contamination of PM by the other membrane fraction which includes the ER. The whole cell extracts were used to measure the total GLUT1 in cells.

### Analysis of the levels of Myc-GLUT1 on the PM

The pLPCX-Myc-GLUT1 vector expressing the GLUT1 with Myc tag in the first exofacial loop was constructed as previously described [32]. The levels of Myc-GLUT1 on the cell surface and in whole cells were measured by IF staining in a flow cytometer as described [32, 41, 42]. To measure the levels of Myc-GLUT1 on the cell surface, at 48 h after cells were transduced with pLPCX-Myc-GLUT1 vectors, cells were blocked in PBS with 2% FBS and stained with a Myc antibody (Roche) to detect Myc-GLUT1 on the cell surface in a flow cytometer To determine the total levels of Myc-GLUT1 in the whole cells, cells were fixed with 2% paraformaldehyde and permeabilized with 0.2% Triton X-100 before staining. The relative levels of Myc-GLUT1 on the cell surface were calculated after normalization with the total levels of Myc-GLUT1 in cells. Cells transduced with empty pLPCX vectors were used as a negative control.

### Immunofluorescence staining

Immunofluorescence (IF) staining of cells was performed as previously described [32]. In brief, cells cultured on coverslips were washed with ice-cold PBS and fixed with methanol. Cells were permeabilized with PBS containing 0.2% Triton X-100. The cells expressing Myc-GLUT1 were incubated with anti-myc antibody (9E10, Roche) overnight followed by Alexa Fluor® 488-conjugated goat secondary antibody (Invitrogen) for 1 h. The coverslips were mounted in Vectashield (Vector Laboratories) and examined by a confocal laser-scanning microscope.

### Chromatin immunoprecipitation (ChIP) analysis

Assays were performed as previously described [37, 43]. The A549-conshR and A549-p53shR, cells were treated with hypoxia (for 18 h) to activate p53 before ChIP assays. The anti-p53 antibody (DO-1, Santa Cruz) and mouse IgG (Santa Cruz) were employed. The sequences for the primer sets used to amplify the DNA fragment containing the potential p53 binding element (p53 BE) in the RRAD promoter: 5'-GTCTACAAAATGGGAACAACAAAT-3' and 5'-TGGGGGCAGGATGATGA-3'. The non-specific (NS) DNA fragment in the RRAD promoter (−1522 to −1244) which does not contain any potential p53 BE was used as a negative control. The sequences for the primer sets for the NS fragment: 5'-CTCCCACCCCCACCACACTTG-3' and 5'-CCAGCAGGAGCCAGCCACTTC-3'. The p53 DNA binding element in human MDM2 promoter region was used as a positive control for ChIP assays [37].

### Quantitative real-time PCR assays

TaqMan Real-time PCR assays were performed as previously described [37]. For RRAD expression analysis in lung tumor samples, RRAD mRNA levels were measured in 24 pairs of human lung cancer samples and their matched adjacent non-tumor lung tissues by using TissueScan lung cancer tissue qPCR panels (Origene, Rockville, MD) by real-time PCR assays. The expression of genes was normalized to the Actin gene.

### Statistical analysis

All *P* values were obtained using a student *t*-test. #: *p*<0.05; *: *p*<0.01.

## SUPPLEMENTARY MATERIAL AND FIGURES


