# Supplementary material for: Tumor suppressor p53 negatively regulates glycolysis stimulated by hypoxia through its target RRAD
Source: Oncotarget. 2014 Jun 26;5(14):5535–46. doi: 10.18632/oncotarget.2137 (PMC4170611; doi:10.18632/oncotarget.2137)
Supplement: Supplementary file 1 [file oncotarget-05-5535-s001.pdf]

Tumor suppressor p53 negatively regulates glycolysis stimulated by hypoxia through its target RRAD

Supplementary Material

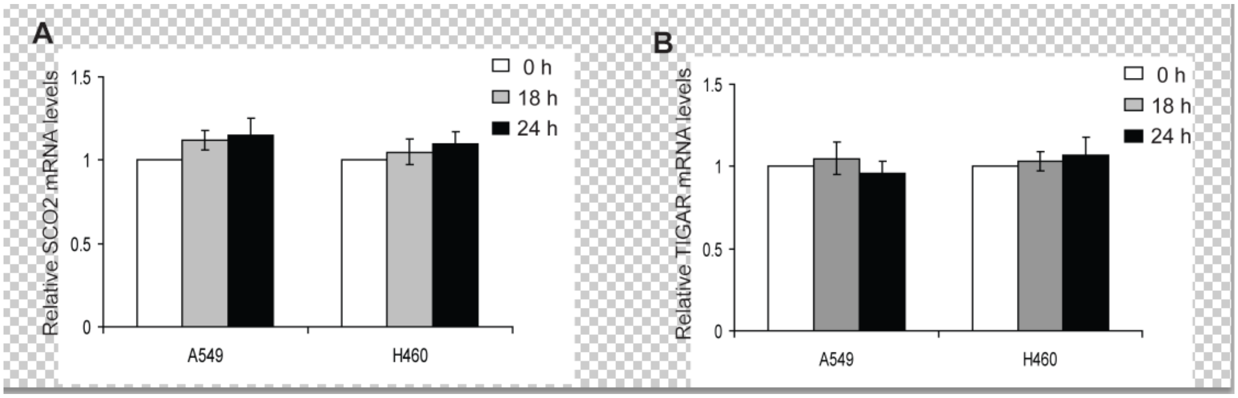

Figure S1. The effect of hypoxia on the expression of SCO2 and TIGAR in A549 and H460 cells. (A) The relative mRNA levels of SCO2 in A549 and H460 cells. (B) The relative mRNA levels of TIGAR in A549 and H460 cells. p53 wild-type A549 and H460 cells were treated with hypoxia for 18 or 24 h. The mRNA levels of RRAD were measured by Taqman real-time PCR assays and normalized with actin. Data are presented as mean value ± SD (n=3).

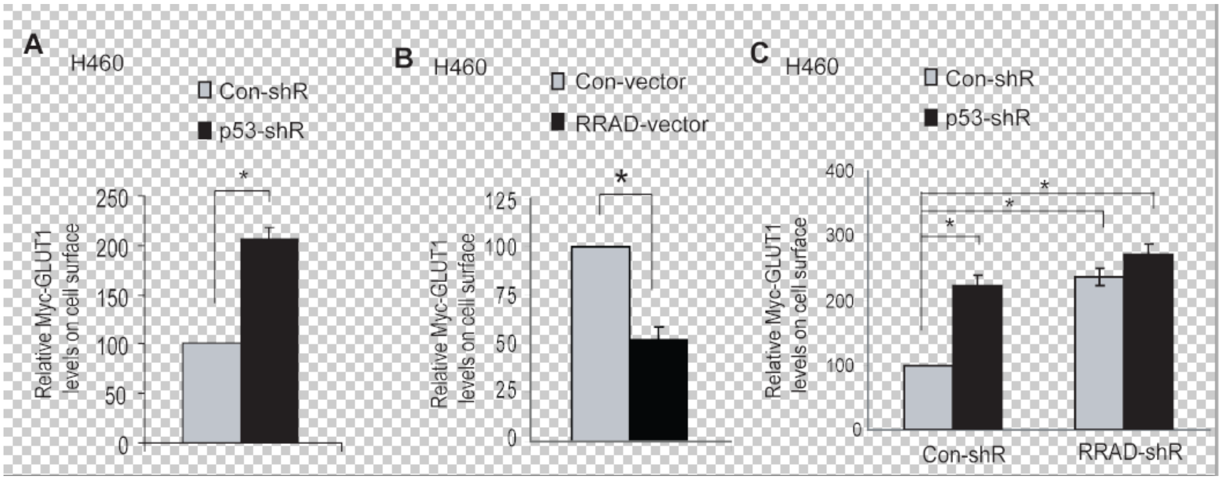

Figure S2. p53 negatively regulates GLUT1 translocation to the plasma membrane through RRAD under hypoxic conditions in H460 cells. (A) p53 reduced Myc-GLUT1 translocation to the plasma membrane stimulated by hypoxia in H460 cells. H460-conshR and H460-p53shR cells were transfected with Myc-GLUT1 vectors and then treated with hypoxia for 24 h before assays. The levels of Myc-GLUT1 on the cell surface were detected by a flow cytometer, and normalized with the total levels of Myc-GLUT1 in cells. (B) Ectopic RRAD expression reduced Myc-GLUT1 translocation to the plasma membrane stimulated by hypoxia in H460 cells. H460 cells with stable RRAD overexpression (RRAD) or control cells (Con) were transfected with Myc-GLUT1 vectors and then treated with hypoxia for 24 h before the levels of Myc-GLUT1 on cell surface were measured by a flow cytometer. (C) RRAD mediated the function of p53 in negative regulation of GLUT1 translocation to the plasma membrane under hypoxic conditions in H460 cells. H460-con-shR and H460-p53-shR cells were transduced with shRNA against RRAD or control shRNA, followed by hypoxia treatment for 24 h. The levels of Myc-GLUT1 on cell surface were normalized to the levels of total Myc-GLUT1 in cells. Data are presented as mean value ± SD (n=3). * $p<0.01$ (Student's *t* tests).
